# Evaluation Method of Financial Accounting Quality in Colleges and Universities Based on Dynamic Neuron Model

**DOI:** 10.1155/2022/8520576

**Published:** 2022-04-21

**Authors:** Lu Liu

**Affiliations:** Shandong Sport University, Jinan, Shandong 250102, China

## Abstract

With the deepening of reform and opening up, great changes have taken place in the university financial management system. The role of financial analysis in university activities is becoming more and more obvious. In the new environment, especially in university financial reporting, we must establish effective reasonable and scientific financial analysis index system and quality evaluation team. In order to reflect the financial situation of colleges and universities, the university financial analysis indicators in this field have important theoretical and practical significance, such as finance, budget implementation, effective utilization of funds, risk prevention, and the formulation and application of such indicators. The financial management level of colleges and universities is improved, and the scientific development of colleges and universities is promoted. In this paper, we introduce the dynamic model of neurons, design a learning algorithm, and apply it to the quality evaluation of financial reports in colleges and universities. Through this research, a single-layer feedback network capable of fast learning and learning is established. This is not only helpful for universities to evaluate the quality of financial accounting business. However, enriching the significance of financial management in higher education has theoretical value.

## 1. Introduction

**Figure 1:**
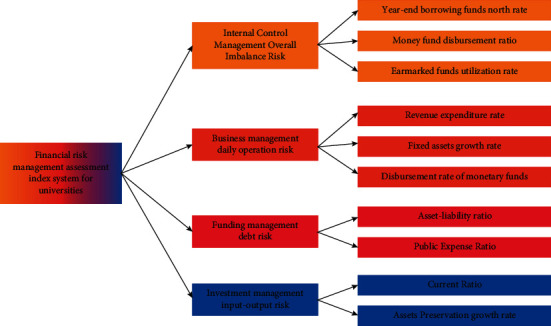
Financial accounting work system in colleges and universities.

Financial analysis of colleges and universities is an important means to improve the efficiency of capital use and management level, an important indicator to promote the school to improve the financial risk tolerance, and an important basis for the school leaders to understand the current situation and make decisions for the future, so it has put forward higher and more requirements for the financial analysis of college system, and it has become vital to actively explore the way to optimize the financial analysis of colleges and universities [1–3]. The connotation of financial analysis of college system refers to the systematic analysis, comparison, and research of business activities of colleges and universities within a certain period of time by using special methods based on and starting from business plans, accounting statements, and other relevant information and summarizing and evaluating the experience of financial management, revealing the existing problems, gradually understanding and grasping the laws of economic activities and business development status of schools, improving the financial management work, and enhancing the financial management level. The importance of studying the optimization measures of financial analysis of colleges and universities is an important part of financial management of colleges and universities. Studying the optimization measures of financial analysis of colleges and universities can provide a more correct and comprehensive understanding of the financial management activities of colleges and universities, grasp the input and use of various resources such as human, material, and financial resources of colleges and universities, analyze the deficiencies in the financial management of colleges and universities, use financial funds more efficiently, and improve the financial management level. In addition, the study of financial analysis optimization measures of colleges and universities can better help financial departments, external investors of colleges and universities, and leaders of colleges and universities understand the financial situation and development trend of colleges and universities and provide factual basis for scientific [4, 5] decision-making. The current situation and problems of financial analysis in college system include the current situation of financial analysis awareness, the current situation of financial analysis content, the current situation of financial analysis methods, the current situation of financial analysis indexes, etc. With the current situation of financial analysis awareness and existing problems, the leaders do not pay enough attention to it, and the focus of attention is not on the financial analysis work on the one hand, most of the school leaders are education connoisseurs but not financial experts, they know about financial knowledge but not expertise, and they do not know enough about financial analysis and do not pay enough attention to it. On the other hand, the financial leaders of colleges and universities focus on the finance department's work of frequent reimbursement (accounting), tuition collection, budgeting, and declaration of spending and lack the initiative and enthusiasm for financial analysis. In the eyes of most school leaders, financial analysis is to analyze and integrate the data in the financial statements, calculate the relevant ratios and related indicators and, at most, summarize the financial situation with textual explanations. The leaders of colleges and universities do not pay enough attention to it, so it leads to the fact that there are few special financial analysis positions in the financial department of colleges and universities to focus on financial analysis, and it is a common phenomenon that financial staffs write down financial reports part-time every year and make simple and rough analysis and integration according to the report data, which does not achieve the real effect of financial analysis and makes the truthfulness and value of accounting information quality of majesty greatly reduced. The quality of financial analysts is not high on the one hand; the focus of the daily work of the financial personnel in colleges and universities lies in bookkeeping, accounting, and reporting, etc. A lot of energy is put into daily reimbursement work and little financial analysis of the economic activities of colleges and universities, and the focus of the financial personnel themselves is not on financial analysis. On the other hand, financial analysts should not only master down-to-earth professional skills, but also have certain management knowledge and understand statistics [6], probability theory, computer, network, and other aspects of information, and of course, they should be very familiar with the situation of this unit, which puts forward high requirements on the quality of financial personnel. The influence of mutual competition among colleges and universities is fierce. In order to stand out in the competition, schools have to develop continuously, expand the scale of enrollment, and build administrative buildings, teaching buildings, canteens, dormitories, gymnasiums, and other infrastructures, which is not enough only by financial funds and income from educational undertakings. In order to promote the rapid development of the school, attract external investors to invest in the infrastructure construction of the university, and obtain financing in many aspects, the financial analysis of the university system becomes very important. The structure of the index system at all levels of financial risk management assessment of universities is shown in Figure 1.

With the in-depth development of China's university system reform, universities gradually rely on financial analysis in behavioral decision-making, and financial management gradually changes from accounting management to analysis management, and financial analysis plays an increasingly prominent and important role in university management [7–9]. However, in the practical application, there are still many problems in the establishment of indicators and quality of analysis in the financial analysis of universities, which need to be improved continuously. Therefore, it is important to find out the problems in financial management through financial analysis, grasp the law of economic operation, and judge the risk level scientifically, so as to improve the financial management level of universities and promote their scientific development. In this paper, we analyze the design and application of financial analysis indexes of colleges and universities under the new situation, establish a scientific and reasonable financial analysis system, and apply it in order to improve the financial management level of colleges and universities and promote the long-term and rapid development of colleges and universities. Currently, under the influence of planned economic management mode, financial analysis has not attracted enough attention in the financial management of colleges and universities in China, and the management concept of financial analysis is not strong. In financial management, colleges and universities still generally focus on the process of nuclear management and supervision, while lacking postevent analysis and forecast. Even if quarterly or annual analysis is done, it is only an after-the-fact analysis. The financial analysis index system is not sound and perfect, and most colleges and universities do not extend the financial analysis index to the design process, not to mention establishing an efficient analysis model and using special analysis tools, thus failing to provide effective financial information for college behavior decision, not to mention adapting to the needs of rapid development of colleges and universities. At present, there are mainly the following problems in the application of financial indexes in colleges and universities: only simple data listing, lack of systematic analysis and scientific methods; the analysis index system is not comprehensive, and the comparability of relevant data is poor; the design of analysis system is not scientific and lacks accuracy, cannot be quantitative analysis, and is not strong in prediction. Therefore, with the deepening of university reform, China has developed higher education as an industry [10]. Under the new situation, it is especially necessary to carry out scientific design of financial analysis indexes and apply them reasonably. The scientific nature should fully consider the characteristics and nature of the management object and its movement law when designing the financial analysis indexes. The indicators are accurate and complete, neither duplicated nor omitted, independent of each other, and complementary to each other. The purpose of effective financial management is to maximize the economic and social benefits of funds by strengthening management. Financial analysis is for the service of financial management, so the design of financial analysis indexes should be centered on improving efficiency. Practicality the financial analysis index system of universities is a complex system with many contents and complicated relationships. When designing the analysis indexes, we should focus on each of them according to the evaluation objectives and tasks, so that the evaluation results of these indexes have practicality. The financial indicators established by comparability should focus on the interconnection, comprehensive, and developmental view and comparability and avoid one-sidedness, so as to draw correct analysis conclusions. Dynamic accounting and education management are constantly changing with the economic situation, and the design of financial analysis index system should also be updatable. If we do not consider from the dynamic nature, we cannot eliminate the influence of chance factors and lose the meaning of analysis and evaluation [10]. Reasonable determination of the weight of each financial analysis index and its ratio of colleges and universities makes the weight and ratio of each index according to the relevant requirements of the department and combined with the actual situation or according to the needs of their own management and development. The standard ratio of financial indicators of universities in recent years can be calculated by referring to other institutions to determine the specific weighting ratio of our indicators or by using arithmetic average. Reasonably determine the upper and lower limits of each single index score. Relevant authorities or universities should make reference to the weight and standard ratio in the financial analysis index system of universities to set the upper and lower limits according to their own situation when formulating analysis indexes, and universities should not be lower or higher than the upper limit when conducting financial evaluation. Reasonable determination of financial analysis standards of colleges and universities is carried out through reasonable analysis after the standards are formulated, and after the analysis, excellent, good, qualified, and unqualified are derived, and the results of financial evaluation of colleges and universities can be obtained by substituting them into the relevant score intervals, which are clear and operable.

Dynamic neuron is an optimization method developed in recent years. It adopts the basic idea and structure of dynamic programming and draws on the achievements and methods of artificial neural networks, computer simulation, artificial intelligence, and other fields to optimize the system itself and improve the performance of the system through the simulation of the actual system. It adopts computer simulation and function approximation [11], which can effectively solve the long-standing “dimensional crisis” and has a broad application prospect in manufacturing, communication, logistics, and control engineering fields. Dynamic neuron is a novel artificial neuron model, whose supervised learning aims at stimulating a string of dynamic neurons that express specific information through precise temporal coding, hence the term dynamic neuron learning. The existing dynamic neuron learning methods are reviewed and compared for the significant application value of single-neuron dynamic neuron learning, various theoretical bases, and many influencing factors. The basic concepts of dynamic neuron model and dynamic neuron learning are first introduced; then the typical dynamic neuron learning methods are introduced in detail, and the theoretical basis and synaptic weight adjustment of each method are pointed out; finally, the performance of these learning methods is compared experimentally, and the characteristics of each method are summarized systematically. The results of this study contribute to the comprehensive application of dynamic neuron learning methods [12].

At present, most universities' financial risk identification mainly relies on managers' experience judgment, which is highly subjective and the accuracy varies from person to person, and it is not easy to discover potential financial risk problems, and it also encounters bottlenecks in promoting the use and publicizing the identification results. Therefore, it is necessary to study a set of financial risk identification models for universities. At present, domestic and foreign scholars have established four main models in financial risk identification: univariate model, multivariate linear discriminant analysis, logistic regression model, and artificial neural network model, which still have shortcomings [13]. The dynamic neuron model has become a hot spot for research because it has no hypothesis requirement for samples and the model has strong fault tolerance, learning ability, and error correction ability, especially in the field of financial risk early warning of listed companies, which has made great progress. However, the research on the quality assessment method of financial accounting work of universities with dynamic neuron model is still in the initial stage. Therefore, this study tries to establish a set of index system for the quality assessment of financial accounting work in colleges and universities from the characteristics of colleges and universities and construct a financial risk identification model for colleges and universities based on dynamic neuron method to achieve the purpose of monitoring and assessment of financial risks in colleges and universities. The main contributions of the model proposed in this paper are as follows:

1. Starting from the characteristics of colleges, this paper establishes a set of quality evaluation index system of financial accounting in colleges and universities
2. This paper constructs a university financial risk identification model based on dynamic neuron method, which has a higher recognition rate than traditional machine learning algorithm and static neural network method
3. The experimental results show that this model can monitor and evaluate the financial risk of colleges and universities, which lays a foundation for relevant research

## 2. Related Work

### 2.1. Financial Accounting in Higher Education

**Figure 2:**
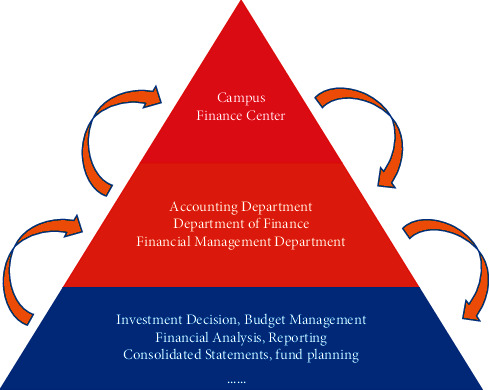
University financial system.

Financial analysis is a quantitative reflection of financial data, often only the amount of revenue, expenditure, budget execution rate, and other data, but with the lack of consideration of the impact of changes in revenue, resulting in low budget execution rate, the “quality” of economic operation is not enough to explore the connotation of this emphasis on quantity. This kind of analysis that emphasizes “quantity” but not “quality” inevitably leads to a lack of foresight and guidance in financial work. The financial analysis is not only to conduct microanalysis of our internal finance, but also to conduct horizontal comparison with other institutions of the same level and carry out macroanalysis. The financial analysis of many institutions is based on the description of accounting statement data, the data description of the basic situation of income and expenditure, and the lack of comparison with the direction, amount, and magnitude of the increase or decrease of the relevant indicators of schools of the same scale of operation or schools with similar operation characteristics, the lack of understanding of the financial situation of the school and the trend of teaching changes, and the lack of reasonable evaluation of their competitive advantages [14]. Neglect of predictive analysis of the results of colleges and universities at the end of economic activities and then regularly has seriously restricted the development of colleges and universities. Colleges and universities should pay more attention to the prior analysis to avoid the drawbacks in the previous work. Otherwise, with the deepening of the reform of college running system, it is difficult for the colleges and universities with unreasonable allocation of educational resources and unsharp professional characteristics to transform into application-oriented universities. Under the new situation, the financial information required for the survival and development of colleges and universities must rely on financial analysis to provide in advance and reduce the competitive pressure of colleges and universities. Neglecting risk factor analysis: Although colleges and universities are nonprofit organizations, they are also in the market economy environment, where the risks and opportunities of running schools coexist and the inputs and benefits go together. Therefore, the financial analysis of colleges and universities only stays on the analysis of some deterministic factors, which has significant hidden dangers. Therefore, colleges and universities should strengthen financial management and strengthen financial risk analysis to prevent major decision-making mistakes from occurring. With lack of cost accounting analysis at present, the economic operation of colleges and universities mainly relies on the income and expenditure standards stipulated in the budget to control the economic activities of each department, so that each department can make full use of educational resources while achieving the set goals. Although the budget control makes the cost expenditure strictly restrained, it ignores the actual situation of various consumptions in economic activities. Therefore, colleges and universities should collect and allocate all kinds of consumptions that occurred in the process of education and teaching, so that the budget and cost can be effectively connected. At the same time, it should also implement the responsibility system for the value preservation and appreciation of state-owned assets and economic cost efficiency indicators and try out the economic responsibility system in a planned way. However, the cost accounting control of colleges and universities is not perfect, the consumption of common resources cannot be measured, teaching work or nonteaching work is costed according to uniform standards, resulting in the lack of authenticity of cost accounting, the educational resources cannot be optimally allocated, and it is difficult to control the phenomenon of occupying and idling resources. Paying attention to the analysis of financial development potential, as a nonprofit institution, the budgeting principles of universities are to balance income and expenditure, keep expenditure within the limits of revenues, and strictly prohibit overspending. The preparation and execution of school budget should be carried out under this principle, and the red line of “operating in debt” and “deficit budget” should not be touched. Under the situation, it is one of the important indexes in the financial performance assessment of colleges and universities to measure the ability of colleges and universities to bear liabilities and risks and to comprehensively assess and evaluate the financial development potential [14]. The specific contents of the appraisal should include the following aspects: the status of accumulated external liabilities of colleges and universities and the ratio of liabilities, the ratio of annual total income and expenditure of schools, the net balance of deposits at the end of the year, the size of lending and temporary payments and their proportion, the assessment of business risks of school-run industries and the ratio of assets and liabilities, etc. In the new era, the essential task of colleges and universities is to cultivate application-oriented talents, meanwhile, strengthen scientific research and transformation of scientific research achievements, and adapt to the development trend of integration of production, learning, research, and application. Therefore, the performance appraisal of colleges and universities cannot be simply equated with the simple economic benefit appraisal but needs to be combined with various achievement appraisals after quantifying the work of colleges and universities as well as the simple economic benefit appraisal of less investment and more production, replacing the simple benefit appraisal with performance appraisal, and summarizing and analyzing the social and economic benefits with performance indexes. The analysis of university running efficiency is a comprehensive work with the characteristics of science and complexity. When analyzing and evaluating based on the above-mentioned indicators, the staff must integrate relevant statistics, funding arrangements, financial plans, fixed indicators, operations, and other relevant information, carefully sort out, compare, and study and analyze all economic activities and achievements of the university, and follow the principle of comparability and make precise calculations for each analysis indicator of the relevant year. Based on this, we compare and analyze the efficiency indicators between different years to reflect the dynamic changes of the efficiency of the university. Improvement of budget expenditure execution analysis of the expenditure budget of colleges and universities includes the expenditure for carrying out teaching, scientific research, management, and other activities in the year. From a macroperspective, the expenditure budget of colleges and universities can be divided into basic expenditure budget and project expenditure budget. If further subdivided, the basic expenditure budget, for example, can be further divided into the budget for personnel expenses and the budget for daily utility expenses. Among them, the budget of personnel expenses can be further divided into salary and welfare expenses, subsidies to individuals and families, including basic salary, allowance, social security contribution, performance salary, and other salary and welfare expenses; the budget of daily public expenses includes office expenses, printing expenses, consulting expenses, utilities, post and telecommunications, heating expenses, and property management expenses. If the university has implemented the operation mode of “group management,” the above detailed item budget can be further subdivided, and the detailed unit budget can be listed by responsible unit. Analyzing the budget execution rate of each department and preparing the analysis table of expenditure budget execution is an effective way to analyze the execution of university expenditure budget. Taking into account the characteristics and actual situation of colleges and universities, when analyzing the budget execution of each expenditure, it is necessary to analyze and evaluate both the absolute number of overspending or saving of each item and the relative number of the degree of budget execution of each funding item. For the budget execution of each expense item, the university should analyze and evaluate the above two aspects comprehensively to get an objective and fair analysis and evaluation result. At the same time, when analyzing the budget execution of expenditure, we should pay attention to the following contents: the data analysis from the budget execution rate is one-sided, not because the budget execution rate is low, the work of saving and use of funds is good, the budget execution rate is high or the expenditure exceeding the budget is the bad work of saving, which will cause the inaccurate results of budget execution of expenditure, and universities need to evaluate the overspending or saving of each item of funds according to the actual situation of using funds. The financial system of the university is shown in Figure 2. The universities need to evaluate the overspending or saving situation of each expense item according to the actual situation of expense use [15].

Colleges and universities usually use the comparative method and ratio method to analyze and compare the income and expenditure structure of each year, so as to compare the similar indicators with those of institutions with the same scale or similar structure in the same period, compare the differences of indicators, and explore the influencing factors that cause the differences, and make objective and scientific financial analysis. Among them, there are three kinds of comparative analysis methods mainly used by colleges and universities: using expenditure budget as the base, comparing the actual expenditure of funds with the budget, and preparing the corresponding budget expenditure execution table; comparing the current period with the same indicators of the previous period, or comparing the current year with the indicators of the same period of the previous year; level comparison, comparing and measuring the same indicators against similar institutions, and finding out the gaps through comparison and comprehensive thinking. To analyze the trend of financial appropriation income and expenditure of colleges and universities, we use the values of various indicators of financial income and expenditure of a certain period as a fixed base period and compare and analyze the data of financial income and expenditure of other periods or other years with them. Through the analysis of the trend of increase/decrease and magnitude change of the indicators in each period compared with the base period, we can trace the reasons for the fluctuation of the indicators, check the financial accounts, judge the financial situation, determine the economic operation and development trend, and predict the possible development results. The indicators with large fluctuations are one of the situations that the trend analysis method focuses on; i.e., it focuses on strengthening the changes of the indicators of special and key parts. By analyzing the trends of the indicators, it is possible to make a general evaluation of the financial income and expenditure and provide a basis for the management to make decisions. The dynamic analysis method takes the quantitative characteristics revealed by the income and expenditure of colleges and universities as the standard, takes the rate of change of economic variables in the process of change in the time series as the basis of analysis, considers the process of change of economic operation from the process of continuous time points, recognizes the law of economic activities, explores the trend of change of economic variables, and judges whether it meets the requirements of strategic development of the university. The fundamental purpose of overall analysis method of financial management is to make limited funds produce greater social and economic benefits. Therefore, when applying the overall analysis method of university finance, it is necessary to follow objective laws, grasp the principle of overall optimization, and pay attention to timeliness, efficiency, and comparability. In this process, colleges and universities should clarify the purpose and take “maximizing efficiency” as the purpose of school economic construction.

### 2.2. Dynamic Neuron Model

Dynamic neural networks are known as the third generation of neural networks, which operate in a manner closer to real neurons and have stronger performance than traditional neural networks. With pulse excitation time as input and output, dynamic neurons can better simulate biological neurons and thus can be the basis for more effective simulation of artificial intelligence [16]. In the research of artificial neural networks, supervised learning theory has been a central part of the research. Since the physiological basis of various cognitive activities is the sequence of impulses excited by neurons in the human brain, supervised learning of dynamic neurons and networks has an important application and has become an extremely important part of its research field. Among them, supervised learning with temporal encoding of the excitation of impulses has become a key research direction for researchers to focus on. The supervised learning methods based on temporal encoding of dynamic neural networks can be broadly classified into single-pulse learning and multipulse learning methods according to the number of pulses excited. Obviously, multipulse learning is more in line with the characteristics of biological neuron operation and also has stronger application performance. Supervised learning methods for dynamic neural networks can be classified into single-neuron learning and network learning methods according to the applicable network structure. A general rule for supervised learning methods in terms of network structure and number of pulses is that single-neuron learning methods are generally more capable of learning multiple pulses and can control more pulse excitation moments through learning, while multilayer network learning methods generally control a smaller number of output pulses. A few definitions of the concepts are introduced here first. Supervised learning of dynamic neural networks refers to learning that causes the output neurons to excite a specific sequence of desired output pulses. Time-coded supervised learning of a dynamic neural network means that the output neurons are learned to excite pulses exactly at the specified moment during a period of operation. Time-coded multipulse supervised learning of single dynamic neuron (hereafter referred to as single-neuron learning) refers to a single dynamic neuron with multiple input synapses that is learned to excite multiple pulses at the desired moment, i.e., a sequence of pulses, so it is also referred to as pulse sequence learning in [16]. Dynamic neurons are the basic units that constitute dynamic neural networks, and supervised learning of single dynamic neurons is of great importance as it can better draw on the well-defined synaptic tuning mechanisms of biological neurons, as well as being the basis for implementing supervised learning of more complex neural networks. The process of single-neuron pulse sequence learning is affected by many factors, including the length of the input and output pulse sequences, the excitation rate, and the number of neuronal synapses. The longer the sequence and the higher the excitation rate, the more difficult the learning process; however, many factors also make the pulse sequence learning more flexible with the available information for weight adjustment. Several single-neuron learning methods have been proposed by different researchers. Among them, one class has been proposed based on stochastic models, and such nondeterministic methods are difficult to analyze and compare their learning ability for complex target sequences [17–19]. An impulse sequence learning method is given using a linear algebraic approach, but the theory of this method is too much detached from dynamic neurons and therefore has received less attention from researchers. Also, there has been a review literature on learning methods for dynamic neural networks, but these studies have been presented more broadly and have not specifically focused on impulse sequence learning methods or conducted experiments to compare the sequence learning ability of different methods. Sequence learning ability is the core performance of pulse sequence learning methods, while methods with stronger learning ability for longer and more complex pulse sequences will have stronger applications and better reflect the characteristics of dynamic neurons.

## 3. Methods

This section describes in detail the quality evaluation method of university financial accounting based on dynamic neuron model. Firstly, it introduces the specific evaluation indexes of university financial evaluation and then introduces the basic principle of dynamic neural network and how to apply it to university financial evaluation.

### 3.1. Evaluation Metrics

**Figure 3:**
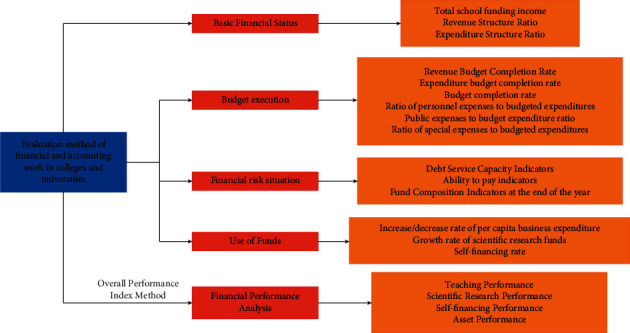
Evaluation indicators.

The index design of financial status analysis of colleges and universities is the performance status of assets, liabilities, and their equity of colleges and universities at a certain point in time. The main indicators are (1) Net asset growth rate = Net asset growth of the current year/Net asset amount of the previous year *∗* 100%. It reflects the efficiency of the university's expenditure and the ability to preserve and increase the value of assets. (2) Ratio of fixed assets formation in current year's expenditure = total capital expenditure in current year/amount of credit incurred in current year's fixed fund *∗*100%, reflecting the capacity of fixed assets and the ability to preserve the value of fixed assets. (3) Growth rate of fixed assets = Current year's fixed fund growth/Prior year's fixed fund balance *∗*100%, reflecting the growth of fixed assets. Design of budget execution analysis indicators: Budget execution analysis indicators mainly include the difference between final income and expenditure and budget income and expenditure and income and expenditure structure analysis indicators, etc. (1) Revenue budget completion rate = total final school revenue/total budget revenue *∗*100%. This indicator reflects the difference between final income and budgeted income, which facilitates the comprehensive and scientific budget management of universities through analysis. (2) Completion rate of expenditure budget = total final expenditure of the university/total budgeted income*∗*100%. This indicator reflects the difference between final expenditures and budgeted expenditures of colleges and universities. Financial risk situation analysis index designs: The design of financial risk aspect indexes is mainly for risk avoidance. The main ones are as follows: (1) Asset-liability ratio = total liabilities/total assets*∗*100%. This indicator reflects the ability of universities to repay their debts. The funding source of colleges and universities is mainly financial allocation, and the asset-liability ratio should be controlled at a small ratio. (2) Current ratio = current assets/current liabilities*∗*100%. This indicator reflects the ability of current assets of colleges and universities to repay current liabilities when they are due. It is more appropriate to keep the ratio at 200%. (3) Cash ratio = Money capital/current liabilities *∗* 100%. This indicator shows the ability of cash to repay current liabilities. (4) Other receivables to current assets ratio = Other receivables/Current assets ending balance *∗* 100%. Other receivables that have not been collected for more than three years will increase the financial risk of universities. (5) Other payables to current assets ratio = other payables/current assets ending balance *∗* 100%. Take care to avoid financial risk when operating with debt. (6) Loan risk factor = outstanding loan balance (/unrestricted net income *∗* 50% + general fund *∗* 25%): 0.8-1.0 high risk; 0.8-0.6 higher risk; 0.6-0.4 medium risk; 0.4-0.2 low risk; 0.2-0 no risk. Design of indicators for fund utilization analysis: (1) Increase/decrease rate of per capita business expenditure = increase of per capita business expenditure of the current year/per capita business expenditure of the previous year *∗*100%. This indicator mainly reflects the cost of per capita expenditure of universities. (2) Self-sufficiency rate = (education income + operation income + other income) (/education expenditure + operation expenditure) *∗*100%. This index reflects the income capacity and the degree of recurrent expenditure of colleges and universities. (3) Growth rate of scientific research funds = the increase of scientific research funds in this year/scientific research funds in the previous year *∗*100%. This index reflects the ability of universities to serve the society. The evaluation factors of the financial work of colleges and universities are shown in Figure 3.

### 3.2. Dynamic Neuronal Network

The starting point of artificial neural network research is to mimic the information processing mechanism of biological neural networks. As neurophysiological research continues to advance, it is recognized that biological neural systems have dynamic properties, which are manifested in the delayed nature of synaptic connections, transmission of impulses, and stimulated excitation of cell membranes. In order to simulate dynamic functions such as learning, adaption, memory, and recall, and thus to better reflect the dynamic properties of biological neurons, it is necessary to introduce a feedback link in the mathematical modeling of biological neurons.

**Figure 4:**
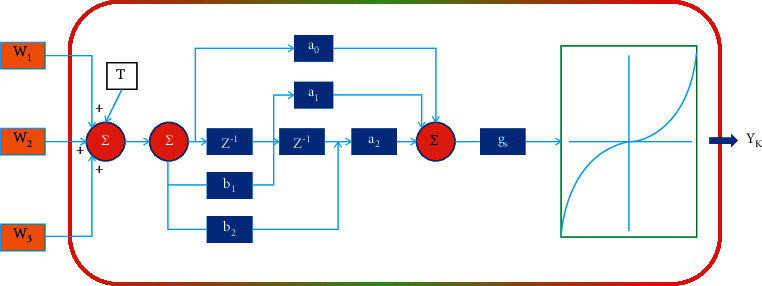
Dynamic neuron model.

Most of the current popular deep networks have the same static reasoning paradigm: once the training is completed, the structure and parameters of the network remain unchanged in the test stage, which limits the representation ability, reasoning efficiency, and interpretability of the model to a certain extent. The dynamic network can adaptively adjust its structure and parameters according to the input samples in the reasoning stage, so it has many good characteristics that the static network cannot enjoy. The dynamic neural unit (DNU) model has both feedforward and feedback weights and then generates output values through the action of nonlinear excitation functions, whose characteristics and structure are different from the traditional static neuron model. The DNU model is shown in Figure 4, where *X*_*i* (*k*) (*i* = 1, 2, ⋯, *n*)∈R ^ n is the input quantity; W_*i* (*i* = 1, 2, ⋯, n)∈R^n are the weights; T ∈ R ^ 1 is the threshold; S(k)∈K^1 is the input of the linear dynamic system; a_ff = [a_0, a_1, a_2 ]^T is the feedforward weight and b_fl = [b_1, b_2]^T is the feedback weight; V_1 (k)∈K ^ (−1) is the output of the linear dynamic system; gs is the slope of the Sigmoid-type nonlinear excitation function curve; V(k)∈R^1 is the input variable of the Sigmoid-type nonlinear excitation function; Y^' (*k*)∈R^' is the output value; *k* denotes a ...rst moment. The linear dynamic system part of the DNU model is essentially a two-order IIR (Infinite Impulse Response) system, which simulates the reverse circuit in the biological nervous system.

The transfer function of the IIR dynamic system part of the DNU model can be expressed as

(1) $$\begin{array}{c} \left(\text{k,a\_ff,b\_fb}\right)=\frac{\left(\text{V\_}1\;\left(\text{k}\right)\right)}{\left(\text{S}\left(\text{k}\right)\right)=\left[\text{a\_0}+\text{a\_}1\text{.}\hat{\text{Z}}\left(-1\right)+\text{a\_}2\text{.}\hat{\text{Z}}\left(-2\right)\right]/\left[1+\text{b\_}1\text{.Z}\left(-1\right)+\text{b\_}2\text{.}\hat{\text{Z}}\left(-2\right)\right]}\text{.} \end{array}$$

Equation (1) can also be equivalently expressed in terms of the difference equation as

(2) $$\begin{array}{c} \text{V\_}1\;\left(\text{k}\right)=-\text{b\_}1\text{.V\_}1\;\left(\text{k}-1\right)-\text{b\_}2\text{.V\_}1\;\left(\text{k}-2\right)+\text{a\_0}.\text{S}\left(\text{k}\right)+\text{a\_}1\text{.S}\left(\text{k}-1\right)+\text{a\_}2\text{.S}\left(\text{k}-2\right)\;\left(2\right). \end{array}$$

From Figure 4, the DNU model treats W_i (*i* = 1, 2, ⋯, n), T,a_i (*i* = 0, 1, 2), b_i (*i* = 1, 2), g_s as adjustable parameters and modifies their values by learning. The learning algorithm can be optimized by using gradient descent method. Since the DNU model treats the feedback weights as adjustable parameters and modifies their values by learning, this inevitably brings the problem of dynamic stability of the model. In this paper, an improved dynamic neuron model, the CIIRDNU (Constricted Infinite Impulse Response Dynamic Neural Unit) model, is proposed. This model limits the feedback weights b_i (*i* = 1, 2)”,” W_i (*i* = 1, 2, ⋯, n), T, a_i (*i* = 1, 2), *g*_s as adjustable parameters to modify their values by learning. The learning algorithm is optimized using the gradient descent method. Since the feedback link variables *b*(*i* = 1, 2) are not modified as adjustable parameters in the model, the stability of the model can be ensured with proper initial settings. If *b*1 = *b*2 = 0, then according to the stability analysis of discrete linear system, *P*1 = *P*2 = 0, the linear dynamic system part of the model becomes FIR system, which can solve the stability problem of IIRDNU model, that is, another improved dynamic neuron model: FIR-DNU (Finite Impulse Response Dynamic Neural Unit) model. In this paper, traditional pattern recognition methods are combined with neural networks to realize the mapping from sample feature pattern space to pattern category space using feedforward networks of dynamic neuron models. The nonlinear excitation function in the dynamic neuron model is taken as

(3) $$\begin{array}{c} \text{Y}\left(\text{k}\right)=\psi \left[\text{V}\left(\text{k}\right)\right]=\frac{1}{\left(1+\hat{\text{e}}-\left(\text{V}\left(\text{k}\right)\right)\right)}\text{.} \end{array}$$

**Figure 5:**
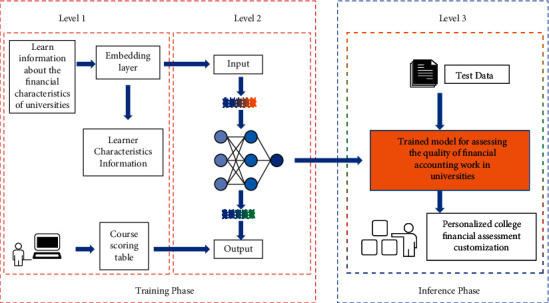
Model architecture diagram.

From equation (3), the node output will be equal to 0 or 1 only when V(k)⟶ ± ∞. Therefore, for the binarized target output, the learning goal is considered to be reached as long as the output reaches values close to 0 and 1 at the time of learning. The overall architecture of the proposed model is shown in Figure 5.

## 4. Results

### 4.1. Dataset

The financial data of 16 universities (SH1-SH16) in 2018 were collected to construct neural network models, and the BP neural network data mining method was applied to analyze the financial situation of 2 of them (SH15 and SH16) in 2018 for early warning.

### 4.2. Experimental Setup

The experimental environment configuration information is shown in Table 1.

The number of neurons in the input and output layers of a BP network is determined by the number of dimensions of the input and output vectors. The dimension of the input vector is also the number of influencing factors. Through the analysis of factors affecting the financial status of universities, the article selects three primary indicators such as solvency, operation capacity, and development capacity and 15 secondary indicators such as asset-liability ratio, so the number of neurons in the input layer is 15. In order to refine the financial risk level, the risk level is divided into five levels: giant alarm, heavy alarm, medium alarm, light alarm, and no alarm, so the target output patterns are (00001), (00010), (00100), (01000), and (10000), which are giant alarm, heavy alarm, medium alarm, light alarm, and no alarm, respectively [2, 5]. This gives the number of neurons in the output layer as 5.

Once the network structure is determined, the sample data is used to train through learning rules to improve the network's adaptive capacity. The learning rate determines the amount of change in the weight Figure Neural Network Modeling data stream values in each cycle and is an important factor in the training process. It is generally preferred to choose a smaller learning rate to ensure the stability of learning, and the learning rate is taken as 0.05 in this paper. The article collects the financial data of 16 universities (SH1-SH16) in 2018 as the research sample, and a total of 14 sets of financial indicators of the first 14 universities (SH1-SH14) in 2018 data and financial risk levels as network training samples, and the financial data of the last 2 universities (SH15 and SH16) in 2018 as prediction samples to apply BP neural network for early warning analysis of university financial risks.

### 4.3. Experimental Results

The results from the network output show that the network achieves the error requirement after 13 training sessions. The network output and the actual risk level of the university are shown in Table 2.

The results of the study show that the BP neural network model has a prediction accuracy of 98%, and the more the important variables for the model, the better the training effect. This shows that the BP neural network successfully forecasts the financial situation of 2 universities (SH15 and SH16) in 2018. The financial risk identification results are shown in Table 3.

### 4.4. Comparative Experiment

Then, the model in this paper is compared with static neural network and SVM classification algorithm. The experimental method is the same as the previous text. The experimental results are shown in Table 4. It can be seen that the recognition accuracy of static neural network and SVM algorithm is 89% and 85%, respectively, which are lower than 98% of the accuracy of this model, which verifies the effectiveness of this model.

## 5. Conclusion

The financial management situation is more a combination of nonfinancial indicators and financial indicators, which can reflect the main measures and effectiveness in terms of budget management, internal control management, asset management, performance management, and talent team building. The description of nonfinancial indicators is an important reference factor to assist financial indicators for trend analysis, which not only is helpful for internal managers to make financial decisions, but also provides important reference information to external supervisors.

Under the new situation, the management system of China's colleges and universities has undergone great changes, and the role of financial analysis in financial management has become increasingly prominent. This paper is practical and valuable for university management by establishing and practically applying the financial index analysis system of universities, and it has certain reference and meaning for other universities to study. However, the financial analysis index system is a dynamic process, which keeps changing with the development of universities. Therefore, the research on the financial analysis index system of universities also needs to be adjusted continuously to meet the development of universities.

In this paper, we will assess the quality of financial accounting work in colleges and universities based on dynamic neuron model. It is studied that feedforward neural network can better reflect the neural computation ability, as long as the appropriate dynamic neuron model is selected (i.e., set the appropriate order n), the single-layer feedforward network established on this basis can solve the problem, and the learning training speed is fast and good. The future research direction is to improve the dynamic neural network from the perspective of structure and cognitive mechanism, so that it can not only maintain high recognition accuracy, but also adapt to complex and unknown data and have stronger robustness. The dynamic neuron model has the potential for applications that are worthy of further research and development. The assessment results are generally consistent with the actual financial situation of the school, indicating that the financial risk assessment index system has a certain degree of reliability and validity [20].

## Figures and Tables

**Table 1:** Software and hardware configuration information.

Project	Configuration
CPU	Intel Core i7 7700
Memory	128G
GPU	NVIDIA GeForce GTX 2080ti
Operating system	Windows 10
Cuda	Cuda_9.0.176_384.81 with cudnn9.0
Language environment	*Python* 3.6

**Table 2:** Model output results with actual value.

Colleges and universities	Year	Actual warning value	Network output
SH15	2018	(01000)	(0.1635 1.0000 0.0002 0.0004 0.0064)
SH16	2018	(00010)	(0.0000 0.0003 0.0043 0.9634 0.0051)

**Table 3:** Financial risk identification results.

	Group 1	Group 2	Group 3
Actual output value	1.2457	2.0121	3.2807
Ideal output value	1.0000	2.0000	3.0000
Relative recognition error	25%	1%	9%
Average recognition error	12%		

**Table 4:** Comparison of experimental results.

	Static neural network (%)	SVM (%)	Our model (%)
Recognition accuracy	89	85	98

## Data Availability

The datasets used during the current study are available from the corresponding author on reasonable request.
